# Root exudate cocktails: the link between plant diversity and soil microorganisms?

**DOI:** 10.1002/ece3.2454

**Published:** 2016-09-23

**Authors:** Katja Steinauer, Antonis Chatzinotas, Nico Eisenhauer

**Affiliations:** ^1^ German Centre for Integrative Biodiversity Research (iDiv) Halle‐Jena‐Leipzig Leipzig Germany; ^2^ Institute of Biology Leipzig University Leipzig Germany; ^3^ Department of Environmental Microbiology Helmholtz Centre for Environmental Research‐UFZ Leipzig Germany

**Keywords:** aboveground–belowground interactions, biodiversity–ecosystem functioning, Jena Experiment, rhizodeposition, soil microbial biomass, soil microbial communities

## Abstract

Higher plant diversity is often associated with higher soil microbial biomass and diversity, which is assumed to be partly due to elevated root exudate diversity. However, there is little experimental evidence that diversity of root exudates shapes soil microbial communities. We tested whether higher root exudate diversity enhances soil microbial biomass and diversity in a plant diversity gradient, thereby negating significant plant diversity effects on soil microbial properties. We set up plant monocultures and two‐ and three‐species mixtures in microcosms using functionally dissimilar plants and soil of a grassland biodiversity experiment in Germany. Artificial exudate cocktails were added by combining the most common sugars, organic acids, and amino acids found in root exudates. We applied four different exudate cocktails: two exudate diversity levels (low‐ and high‐diversity) and two nutrient‐enriched levels (carbon‐ and nitrogen‐enriched), and a control with water only. Soil microorganisms were more carbon‐ than nitrogen‐limited. Cultivation‐independent fingerprinting analysis revealed significantly different soil microbial communities among exudate diversity treatments. Most notably and according to our hypothesis, adding diverse exudate cocktails negated the significant plant diversity effect on soil microbial properties. Our findings provide the first experimental evidence that root exudate diversity is a crucial link between plant diversity and soil microorganisms.

## 1 Introduction

It has long been recognized that biodiversity is not only the result of ecosystem processes, but also an important driver of ecosystem functions itself (Cardinale et al., 2011; Hooper et al., 2012). In aboveground–belowground interactions, plant diversity plays an essential role for ecosystem functioning (Wardle et al., 2001; Zak et al., 2004). Alterations in plant diversity affect aboveground functions (Cardinale et al., 2011), such as plant productivity (Tilman et al., 2015), and have an impact on belowground processes and soil biota (Hooper et al., 2000; Tilman, Reich, & Knops, 2015). Plants affect the soil microbial community through biomass production, litter quality, and belowground carbon allocation and nutrient movements (Dennis, Miller, & Hirsch, 2010; Wardle et al., 2001). Previous studies showed that the biomass and activity of soil microorganisms increase significantly with increasing plant diversity (Eisenhauer et al., 2010; Spehn, Joshi, Schmid, Alphei, & Körner, 1994; Steinauer et al., 2009). Additionally, results from a long‐term grassland biodiversity experiment (the Jena Experiment) have shown that bacterial and fungal diversity increase with higher plant diversity (Lange et al., 2015). Thus, plant community composition greatly influences the community composition of root‐associated organisms (Lange et al., 2015; Mitchell et al., 2010).

Within the rhizosphere, plant roots must compete with neighboring plant roots and soil‐born organisms for space, water, and nutrients (Narula, Kothe, & Behl, 2009). Therefore, plants produce various chemical compounds initiating communication with soil microbial communities to defend themselves against harmful organisms and to attract beneficial microbes (Philippot, Raaijmakers, Lemanceau, & van der Putten, 2013). These compounds include root exudates, defined as soluble low molecular weight components, and comprise different amino acids, organic acids, sugars, and secondary metabolites (Rovira, 2013). Root exudates are used by soil microorganisms for energy supply and biomass production (Baudoin, Benizri, & Guckert, 2003) and thereby clearly represent a major source of soil organic carbon (Marschner, 1995).

The qualitative and quantitative composition of root exudates is determined by plant species, plant age (Hertenberger, Zampach, & Bachmann, 2002; Martin, 1971), and several edaphic factors, including soil type (Neumann et al., 2014) and soil moisture (Boone, Nadelhoffer, Canary, & Kaye, 1998). Most importantly, different plant species release diverse sets of organic compounds that change rhizosphere conditions and affect microbial community structure, abundance, and activity (Sørensen, Haubjerg Nicolaisen, Ron, & Simonet, 2003). For that reason, it is expected that the more diverse the plant community is, the more diverse the composition of root exudates, and consequently the higher soil microbial diversity will be (Philippot et al., 2013). Thus, root exudates may represent the mechanistic link between the composition of the plant community and the composition and functioning of soil microbial communities (Eisenhauer et al., 2013; Lange et al., 2015).

Additionally, plant community and root exudate composition can alter the activity of extracellular enzymes (Kreyling et al., 2008). These enzymes produced mainly by soil microorganisms and, to a lesser extent, by plant roots degrade polymeric substances (e.g., cellulose, lignin) into accessible subunits (e.g., sugar, amino acids) for metabolism and growth of soil microorganisms and plants, respectively (Sanaullah, Blagodatskaya, Chabbi, Rumpel, & Kuzyakov, 1964; Sinsabaugh, 2010). For instance, phenol oxidase depolymerizes lignin and is known to indicate increasing activity of specialized soil fungi and actinomycetes (Keyser, Kirk, & Zeikus, 1978; Kirk & Farrell, 1987), whereas cellulose‐degrading enzyme activities coincide with an increasing dominance of heterotrophic fungi (Lynd, Weimer, van Zyl, & Pretorius, 2002). Thus, studying specific extracellular enzyme activities can provide some insights into the present soil microbial community.

**Figure 1:**
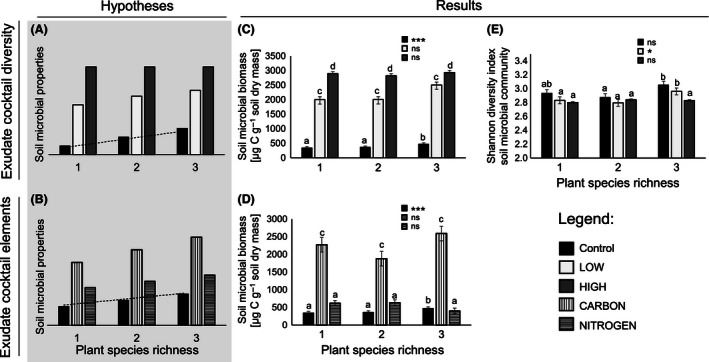
Conceptual figures and experimental results regarding the interdependence of plant diversity and exudate diversity in affecting soil microbial properties. (A) Soil microbial properties (biomass and diversity) are expected to increase with plant diversity (e.g., Eisenhauer et al., 2010; Lange et al., 2015). Dotted line displays the expected significant results. The addition of diverse exudate cocktails is expected to negate plant diversity effects on soil microbial properties. (B) Soil microbial properties were expected to be more carbon‐limited than nitrogen‐limited in the tested soil (Eisenhauer et al., 2010). Dotted line displays the expected significant results. Effects of artificial root exudate diversity on soil microbial biomass [μg C g^−1^ soil dry mass] (in (C): black bars: control; light gray bars: LOW‐exudate diversity treatment; dark gray bars: HIGH‐exudate diversity treatment) (in (D): black bars: control; vertically striped bars: CARBON‐rich exudate cocktail; horizontally striped bars: NITROGEN‐rich exudate cocktail), and (E) Shannon diversity index of soil microbial community. Bars with different letters vary significantly (Tukey's HSD test, α < .05). ***: *p* < .001; *: *p* < .05; ns: *p* > .1. Dotted line

In this study, we explored whether higher root exudate diversity enhances soil microbial biomass and diversity in a plant diversity gradient, thereby offsetting the positive plant diversity effect on soil microbes. We set up a plant diversity gradient ranging from monocultures to two‐ and three‐species mixtures in microcosms using functionally dissimilar plant species in soil of the Jena Experiment, Germany (Ebeling et al., 2014). We applied artificial exudate cocktails to the microcosms combining the most common sugars, organic acids, and amino acids found in exudates (Rovira, 2013). In total, we created four different exudate cocktails, two exudate diversity levels (LOW, 7 compounds; HIGH, 18 compounds) and two nutrient‐enriched levels (CARBON, 98.33% C, 1.67% N; NITROGEN, 76.11% C, 23.89% N), and a control with water addition only. We hypothesized that (i) increasing plant diversity leads to increasing soil microbial biomass (Eisenhauer et al., 2010) and diversity (Lange et al., 2015) and (ii) the addition of a diverse exudate cocktail will negate significant plant diversity effects on soil microbial properties (Fig. 1A). Soil microbial activity is limited by easily accessible organic carbon (Hodge, Robinson, & Fitter, 2000; Schimel & Weintraub, 1992), as also found in the Jena Experiment (Eisenhauer et al., 2010). Therefore, we also hypothesized that (iii) soil microorganisms in the Jena Experiment are more carbon‐limited than nitrogen‐limited, and nutrient limitation is increasing with plant diversity due to higher competition for nutrients between plants and soil microorganisms (Fig. 1B).

## 2 Material and methods

### 2.1 Experimental design

We set up a microcosm experiment using common flower pots (inner diameter: 11 cm; height 9.4 cm) covered by a 5 mm mesh at the bottom to allow drainage of water. Prior to use, the soil was defaunated by three freeze–thaw cycles at −20°C and room temperature (Huhta, Persson, & Setälä, 1998) to exclude any possible grazing and competition effects for nutrients of higher trophic levels, such as Collembola, on soil microbial biomass and diversity. The microcosms were filled with 400 g of sieved (mesh diameter: 2 mm) and homogenized soil (pH 8.1) from the Jena Experiment, Jena, Germany (Roscher et al., 2009). During the experiment, the microcosms were placed in a temperature‐controlled climate chamber (Johnson Controls, Burscheid, Germany) set at 20°C light (16 hr) and 16°C dark (8 hr).

Three functionally dissimilar plant species were grown in separate trays in the same soil as used for the experiment: *Anthoxanthum odoratum* (grass), *Plantago lanceolata* (small herb), and *Centaurea jacea* (tall herb). We set up a plant diversity gradient ranging from monocultures to two‐ and three‐species mixtures. We always planted the same total densities with three plant individuals per microcosm (in the case of two‐species mixtures, we used both possibilities equally of combining one individual of species A with two individuals of species B and *vice versa*).

To test for root exudate diversity effects, we created four different artificial exudate cocktails of primary metabolites combining the most common sugars, organic acids, and amino acids in C_3_‐plants (Vranova, Rejsek, Skene, Janous, & Formanek, 2006). As the function of several secondary metabolites is still not fully understood, we excluded them in the present study, although they might play a significant role in plant diversity effects on soil microorganisms. We produced two exudate diversity levels (LOW‐ and HIGH‐diversity) and two nutrient‐enriched levels (CARBON‐ and NITROGEN‐enriched) (Table 1). The low‐exudate diversity treatment (LOW) contained seven different compounds (Table 1). The high‐exudate diversity treatment (HIGH) contained the same concentrations but 18 different compounds (Table 1). The carbon‐enriched exudate diversity treatment (CARBON) contained nine different carbon‐rich chemical compounds, whereas the nitrogen‐enriched exudate diversity treatment (NITROGEN) contained nine nitrogen‐rich compounds (Table 1). Overall, we set up 105 microcosms (three monocultures, three different two‐species mixtures, and one three‐species mixture × five different exudate treatments (LOW, HIGH, CARBON, NITROGEN, and control) × three replicates) randomly arranged in four blocks to account for potential temperature and light differences within the climate chamber. All compounds were obtained from Carl Roth (Germany).

**Table 1:** Composition of four different artificial exudate cocktails (LOW, HIGH, CARBON, NITROGEN), the total concentration of different sugars, organic acids, or amino acids added each time, the amount of C [μg C g^−1^ soil] and N [μg N g^−1^ soil] added each time, the C:N of each addition, and the total amount of C [g C g^−1^ soil] and N [g N g^−1^ soil] added over the whole experiment

Exudate diversity levels	LOW	Total concentration of … added each time		**Sugars**	40 mmol L^−1^	**Organic acids**	20 mmol L^−1^		**Amino acids**	10 mmol L^−1^		
		Compounds		Glucose	Sucrose	Acetic acid	Succinic acid		Alanine	Glutamic	Glycine	
		C added each time [μg C g^−1^ soil]	710.6									
		N added each time [μg N g^−1^ soil]	23.3									
		C:N added each time	30.5									
		Total C added [g C g^−1^ soil]	2.56									
		Total N added [g N g^−1^ soil]	0.08									
	HIGH	Total concentration of … added each time		**Sugars**	40 mmol L^−1^	**Organic acids**	20 mmol L^−1^		**Amino acids**	10 mmol L^−1^		
		Compounds		Glucose	Fructose	Acetic acid	Citric acid	Lactic acid	Alanine	Arginine	Asparagine	Glutamic
				Sucrose	Maltose	Fumaric acid	Malic acid	Succinic acid	Glycine	Histidine	Leucine	Tyrosine
		C added each time [μg C g^−1^ soil]	732.4									
		N added each time [μg N g^−1^ soil]	30.6									
		C:N added each time	23.9									
		Total C added [g C g^−1^ soil]	2.64									
		Total N added [g N g^−1^ soil]	0.11									
Nutrient‐enriched levels	CARBON	Total concentration of … added each time		**Sugars**	40 mmol L^−1^	**Organic acids**	20 mmol L^−1^		**Amino acids**	10 mmol L^−1^		
		Compounds		Glucose	Fructose	Citric acid	Malic acid	Succinic acid	Leucine	Tyrosine		
				Sucrose	Maltose							
		C added each time [μg C g^−1^ soil]	685.4									
		N added each time [μg N g^−1^ soil]	11.6									
		C:N added each time	58.8									
		Total C added [g C g^−1^ soil]	2.47									
		Total N added [g N g^−1^ soil]	0.04									
	NITROGEN	Total concentration of … added each time		**Sugars**	10 mmol L^−1^	**Organic acids**	20 mmol L^−1^		**Amino acids**	40 mmol L^−1^		
		Compounds		Glucose	Fructose	Acetic acid	Lactic acid		Arginine	Asparagine	Glutamic	Histidine
		C added each time [μg C g^−1^ soil]	480.4									
		N added each time [μg N g^−1^ soil]	175.1									
		C:N added each time	2.7									
		Total C added [g C g^−1^ soil]	1.73									
		Total N added [g N g^−1^ soil]	0.63									

During the first 4 weeks of the experiment, all microcosms were watered every second day with 50 ml of distilled water. Afterward, either 50 ml of the exudate solutions or 50 ml distilled water was added in the center of each pot every second day to assure equal distribution of exudates within the rhizosphere of each plant individual. Thus, exudate solutions were added nine times. Detailed information on C and N input and C:N ratio is listed in Table 1.

### 2.2 Harvest and plant biomass

After 2 months, we stopped the experiment and harvested the plant shoot material by cutting shoots at the soil surface. The entire soil—a mixture of both rhizosphere and bulk soil—of each microcosm was sieved (2 mm mesh size) to remove all roots and determine total root biomass after root washing. Total shoot and root biomass were determined by weighing the samples after drying at 70°C for 72 hr. The sieved soil was divided into two parts; the first part was stored at 4°C for soil microbial biomass measurements, and the second part was immediately frozen at −20°C for a DNA‐based determination of soil microbial diversity (terminal restriction fragment length polymorphism (T‐RFLP).

### 2.3 Soil microbial biomass and diversity

Approximately 4.5 g soil (fresh weight) was used to determine soil microbial biomass using an automated respirometer based on electrolytic O_2_ microcompensation (Scheu, 2002). Soil microbial biomass was calculated from the maximum initial respiratory response (MIRR) after the addition of D‐glucose‐monohydrate using the substrate‐induced respiration method (SIR) (Anderson & Domsch, 1978). Catabolic enzymes of soil microorganisms were saturated by adding 40 mg glucose per g soil dry weight as an aqueous solution. The SIR method might underestimate part of the microbial community (e.g., filamentous fungi), and it has been successfully used to detect significant plant diversity effects on soil microbes and the processes they drive (Eisenhauer et al., 2010; Lange et al., 2015; Thakur, Milcu, & Manning, 2000).

For analysis of soil microbial diversity, we only used samples of the control, LOW‐, and HIGH‐exudate diversity treatment because the root exudate diversity treatments were the main focus of this study. DNA was extracted from 0.5 g of soil with the NucleoSpin Soil Kit Macherey‐Nagel, Düren, Germany following the manufacturer's instructions. DNA quality was checked by agarose gel electrophoresis, and DNA quantity was determined spectrophotometrically (NanoDrop^®^ ND‐1000; Peqlab, Germany). T‐RFLP analysis was performed with 16S rRNA gene amplification products obtained after PCR with a 6‐carboxyfluorescein (6‐FAM)‐labeled forward primer 27f (5′‐AGAGTTTGATCMTGGCTCAG‐3′) and an unlabeled reverse primer 1492r (5′‐GGTTACCTTGTTACGACTT‐3′) (Lane, 1991). PCRs were performed in a reaction volume of 12.5 μl and comprised 2× MyTaq Red Mix (Bioline, Luckenwalde, Germany), 0.01 μmol L^−1^ of each primer, and 6 ng DNA. PCR conditions included a denaturation step at 95°C per 3 min followed by 30 cycles of 95°C per 15 s, 56°C per 15 s, 72°C per 70 s, and finally 72°C per 10 min. PCR products were checked by agarose gel electrophoresis and purified using the cleanup system SureClean Plus (Bioline), and their quantity was determined by gel quantification using GeneTools (Syngene, Cambridge, UK). A total of 75 ng purified PCR product from each sample was digested overnight at 37°C with 2U *MspI* and *HhaI* (New England Biolabs, Germany) in separate reactions and subsequently precipitated with ethanol. T‐RFLP analysis was run on an ABI PRISM 3100 genetic analyzer system using the GeneScan^™^ 500 ROX^™^ size standard (both from Applied Biosystems, Life Technologies, Germany). As described by Giebler, Wick, Chatzinotas, and Harms (2013), T‐RFLP data were analyzed with GeneMapper V3.7 (Applied Biosystems). Briefly, T‐RFs were binned and aligned using the method of Abdo et al., (2006) in statistical software R version 2.10.0 (R Development Core Team 2009). Output tables were translated into presence/absence matrices including information on the relative abundance of each T‐RF detected in the different samples and subsequently used for further analysis.

### 2.4 Extracellular enzyme activity assays

The activities of two soil enzymes were measured to get insights into microbial community function. We chose cellobiohydrolase and phenol oxidase (EC 1.10.3.2) involved in cellulose and lignin decomposition and indirect indicators of fungal activity (Baldrian & Valásková, 2008).

Enzyme activities of cellobiohydrolase were measured using fluorogenically 4‐methylumbelliferone (MUB)‐labeled substrate using 4‐MUB‐β‐D‐cellobioside (EC 3.2.1.91) as substrate. Cellobiohydrolase transforms cellulose into cellobiose and is a crucial component of the C‐cycle. Prior to the enzyme assays, a sample suspension was prepared from 1 g soil and 125 ml of 50 mmol L^−1^ Tris buffer (pH 8.0) and homogenized using an ultrasonic bath for 1 min. According to the pH value of the soil samples, the pH of 8.0 was chosen to maintain natural soil conditions. For fluorimetric enzyme assay (adapted to Saiya‐Cork, Sinsabaugh, & Zak, 2004), we set up four replicates of sample wells with substrate, two replicates of blank wells with buffer, and four negative ambient wells including substrate and buffer, as well as a calibration curve for each sample with standard concentrations of 0, 2.5, 5, 10, and 20 μmol L^−1^ per assay. Substrate concentration was 200 μmol L^−1^. After incubation for 45 min (HIGH treatment) and 3 hr (control and LOW), respectively, at 20°C in the dark, the fluorescence was measured at 365 nm excitation and 450 nm emission using a BMG FLUOstar Omega Reader (BMG Labtech, Ortenberg, Germany). Enzyme activities were then expressed in nano moles per hour per gram of dry soil (nmol hr^−1^ g^−1^).

The absorbance of colorimetric enzyme reaction products of the oxidative enzymes phenol oxidase, involved in lignin degradation, was measured spectrophotometrically using L‐3,4‐dihydroxyphenylalanine as substrate. The absorbance of the oxidative enzymes was determined using 96‐well plates as reported in Saiya‐Cork et al. (2004) including four replicates of sample wells and substrate, four replicates of negative ambient wells, and four replicates of blank wells. Substrate concentration was 25mM (control) and 5mM (LOW and HIGH treatment). The activities of phenol oxidase were measured at 450 nm after an incubation period of 18 hr (control), 4 hr (LOW), and 5 hr (HIGH) in the dark at 20°C using a BMG FLUOstar Omega Reader (BMG Labtech, Ortenberg, Germany). Enzyme activities were then expressed in micromoles per hour per gram of dry soil (μmol hr^−1^ g^−1^).

### 2.5 Statistical analysis

First, we used linear mixed‐effects models to test the effects of plant diversity (PD), treatment (control, LOW, HIGH, CARBON, NITROGEN), and the interaction between PD and treatment on root and shoot biomass, microbial biomass, microbial richness (i.e., number of T‐RFs), Shannon diversity index, evenness, and on both extracellular enzymes (cellobiohydrolase and phenol oxidase). Thereby, “block” was used as a random intercept. Although we found no significant interaction effects between PD and treatment (except cellobiohydrolase), we observed different slopes between plant diversity and root exudate treatments and analyzed the effects of plant diversity on all variables in all treatments separately in order to explore treatment‐specific plant diversity effects. Treatment‐specific analyses of plant diversity effects allowed us to test our hypotheses. We also used linear mixed‐effects models and comparisons of means for treatment‐specific plant diversity effects (Tukey's HSD test; α < .05). *p*‐ and *F*‐values and degrees of freedom were estimated with Type III Satterthwaite approximation for all linear mixed‐effects models. Linear mixed‐effects models and Tukey's tests were performed using lme4 (Bates, Maechler, & Bolker, 2015) and multcomp package (Hothorn et al., 2016) within the R statistical environment (R Development Core Team 2013).

We also used a heatmap visualization of T‐RFLPs with relative abundances and a nonmetric multidimensional (NMDS) analysis to visualize the dissimilarities (based on Bray–Curtis dissimilarities) in microbial community composition. The heatmap and NMDS analyses were run in PAST (3.11) statistical software (Hammer, Harper, & Ryan, 2001). Further, we ran permutational multivariate analysis of variance (PERMANOVA) (based on Bray–Curtis dissimilarities) to test whether differences among microbial communities in the different treatments were significant using the “adonis” function in the “vegan” package (Oksanen et al., 2016).

## 3 Results

### 3.1 Plant biomass

Shoot biomass was not significantly affected by plant diversity (Table 2; Fig. S1). However, shoot biomass was significantly reduced in the LOW (*F*_1,40_ = 30.43, *p* < .001), HIGH (*F*_1,39_ = 8.65, *p* = .005), and NITROGEN (*F*_1,40_ = 79.01, *p* < .001) treatment compared to the control. Root biomass was not significantly affected by plant diversity, but was significantly higher in the control than in the exudate treatments (Table 2).

**Table 2:** Linear mixed‐effects model: table of *F*‐ and *p*‐values for the effects of plant diversity (PD: monoculture and two‐ and three‐species mixtures) and five treatments (Treatment all: Control, LOW, HIGH, CARBON, NITROGEN) on shoot biomass, root biomass, soil microbial biomass, cellobiohydrolase activity, and phenol oxidase activity and of three treatments (Treatment ED: Control, LOW, HIGH) on soil microbial richness, Shannon diversity, and evenness

	Shoot biomass			Root biomass			Soil microbial biomass			Microbial richness			Shannon diversity			Evenness			Cellobiohydrolase			Phenol oxidase		
	*df*	*F*‐value	*p*‐value	*df*	*F*‐value	*p*‐value	*df*	*F*‐value	*p*‐value	*df*	*F*‐value	*p*‐value	*df*	*F*‐value	*p*‐value	*df*	*F*‐value	*p*‐value	*df*	*F*‐value	*p*‐value	*df*	*F*‐value	*p*‐value
PD	2, 89	1.93	.151	2, 90	1.41	.248	2, 85.4	*2.75*	*.070*	2, 51.53	1.96	.152	2, 51.32	2.05	.139	2, 51.36	1.74	.186	**2, 54**	**3.86**	**.027**	2, 54	0.75	.479
Treatment all	**4, 89**	**5.83**	**<.001**	**4, 90**	**11.46**	**<.001**	**4, 86.11**	**173.31**	**<.001**															
Treatment ED										*2, 50.17*	*2.95*	*.064*	*2, 50.17*	*2.95*	*.061*	**2, 50.18**	**4.89**	**.011**	**2, 54**	**388.93**	**<.001**	**2, 54**	**33.29**	**<.001**
PD × Treatment all	8, 89	0.75	.652	8, 90	0.37	.935	8, 86.33	1.58	.143															
PD × Treatment ED										4, 51.32	0.80	.533	4, 51.14	0.92	.460	4, 51.18	2.11	.093	**4, 54**	**5.23**	**.001**	4, 54	0.34	.847

Significant results (*p* < .05) are highlighted in bold, and marginally significant results (*p* < .10) are given in italics.

### 3.2 Soil microbial biomass and genetic diversity (T‐RFLPs)

Soil microbial biomass increased significantly with higher plant diversity in the exudate control treatment (Fig. 1C; Tables 2 and 3) and tended to increase with increasing plant diversity in the LOW treatment. In the HIGH treatment, total soil microbial biomass was similar across all plant diversity levels (Fig. 1C), offsetting the plant diversity effect. Besides, soil microbial biomass in the LOW treatment was significantly higher than in the control (*F*_1,37.26_ = 340.85, *p* < .001) across all plant diversity levels. Additionally, the soil microbial biomass in the HIGH treatment was significantly higher than in the LOW treatment (*F*_1,37.60_ = 65.52, *p* < .001). We found no significant plant diversity effects on soil microbial biomass in the CARBON and NITROGEN treatment (Table 3; Fig. 1D), but soil microbial biomass was significantly higher in the CARBON treatment than in the NITROGEN treatment (Fig. 1D; *F*_1,39.39_ = 84.33, *p* < .001).

**Table 3:** Linear mixed‐effects model: table of *F*‐ and *p*‐values on the effects of plant diversity (monoculture and two‐ and three‐species mixtures) on shoot biomass, root biomass, soil microbial biomass, soil microbial richness, Shannon diversity, evenness, cellobiohydrolase activity, and phenol oxidase activity of each treatment (Control, LOW, HIGH, CARBON, NITROGEN)

	Shoot biomass			Root biomass			Soil microbial biomass			Microbial richness			Shannon diversity			Evenness			Cellobiohydrolase			Phenol oxidase		
	*df*	*F*‐value	*p*‐value	*df*	*F*‐value	*p*‐value	*df*	*F*‐value	*p*‐value	*df*	*F*‐value	*p*‐value	*df*	*F*‐value	*p*‐value	*df*	*F*‐value	*p*‐value	*df*	*F*‐value	*p*‐value	*df*	*F*‐value	*p*‐value
Control	2, 18	*3.18*	*.065*	2, 18	0.52	.603	2, 15.22	**13.04**	**<.001**	2, 17	2.48	.113	2, 17	*2.93*	*.080*	2, 17	2.26	.135	2, 16.67	0.41	.670	2, 15.37	*3.25*	*.067*
LOW	2, 18	1.04	.374	2, 18	0.44	.648	2, 17.26	2.25	.135	2, 17.28	*2.84*	*.086*	2, 16.64	**5.36**	**.016**	2, 17.94	1.57	.235	2, 17.93	0.50	.616	2, 18	0.51	.611
HIGH	2, 17	0.05	.948	2, 18	1.07	.365	2, 18.27	0.89	.430	2, 15.45	0.09	.914	2, 15.40	0.15	.863	2, 15.28	*2.74*	*.096*	2, 18.04	**6.39**	**.008**	2, 18	0.39	.684
CARBON	2, 18	2.29	.130	2, 16.13	0.3	.744	2, 18.26	1.27	.304										2, 17.84	0.23	.800	2, 18	*3.20*	*.065*
NITROGEN	2, 18	0.35	.712	2, 13.65	0.72	.506	2, 17.045	2.58	.105										2, 14.39	0.72	.506	2, 17.56	**0.64**	**.048**

Significant results (*p* < .05) are highlighted in bold, and marginally significant results (*p* < .10) are given in italics.

**Figure 2:**
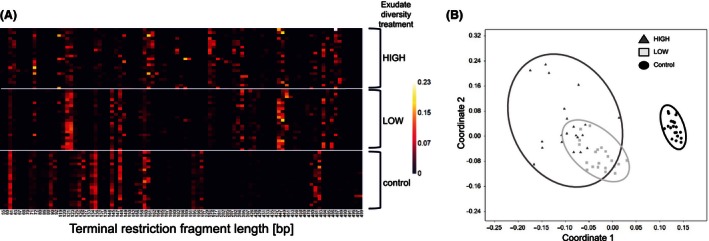
Root exudate diversity effects on soil microbial community composition. (A) Heatmap of relative abundances (median percentage) of terminal restriction fragment length [bp] among samples and treatments (control, LOW‐exudate diversity treatment, and HIGH‐exudate diversity treatment). Colors are scaled from highest (yellow) to lowest (black) values within columns. (B) Nonmetric multidimensional scaling (NMDS) plots based on Bray–Curtis dissimilarities between soil samples with symbols coded by treatment (black circle: control; light gray rectangle: LOW‐exudate diversity treatment; dark gray triangle: HIGH‐exudate diversity treatment). The circles indicate 95% confidence intervals

T‐RFLP analysis revealed no significant effects of plant diversity and exudate treatment on soil microbial richness, the Shannon diversity index, and evenness (Table 2). However, the Shannon diversity index increased marginally significantly with increasing plant diversity in the control exudate treatment and increased significantly in the LOW treatment (Fig. 1E; Table 3) with higher microbial diversity in the three‐species mixture than in the plant monoculture. Shannon diversity was not significantly affected by plant diversity in the HIGH treatment (Fig. 1E; Table 3), again offsetting the significant plant diversity effect on microbial diversity found in the LOW‐exudate diversity treatment. Evenness of T‐RFLP profiles (Table 2) and permutation test revealed significant differences in soil microbial community composition across the different exudate treatments (Fig. 2A, b; *F*_2,61_ = 24.63, *p* = .001), however with a strong overlap between the HIGH and LOW treatment (Fig. 2A,B). However, soil microbial community composition within treatments was not significantly affected by plant diversity (evenness: Table 3; permutation test: *F*_2,61_ = 1.28, *p* = .227). All interactions of plant diversity and exudate treatment on the microbial response variables were not significant (Table 2).

### 3.3 Extracellular enzyme activities

**Figure 3:**
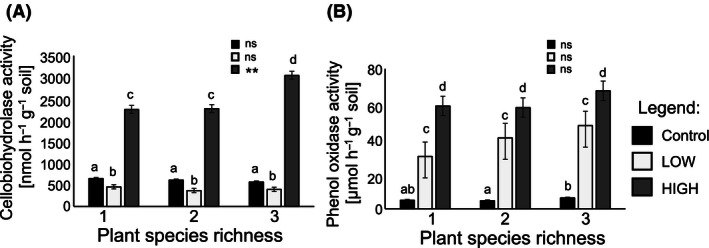
Plant diversity and exudate diversity effects on extracellular enzyme activities. Effects of artificial root exudate diversity on (A) cellobiohydrolase activity [nmol hr^−1^ g^−1^ soil] (black bars: control; light gray bars: LOW‐exudate diversity treatment; dark gray bars: HIGH‐exudate diversity treatment) and (B) phenol oxidase activity [μmol hr^−1^ g^−1^ soil]. Bars with different letters vary significantly (Tukey's HSD test, α < .05). **: *p* < .01; ns: *p* > .1

Cellobiohydrolase activity, indicating the degradation of cellulose, was significantly higher in the HIGH treatment than in the control (Fig. 3A) and significantly lower in the LOW treatment than in the control and HIGH treatments (Fig. 3A). Additionally, enzyme activity increased significantly with increasing plant diversity in the HIGH treatment (Table 3; Fig. 3A).

The activity of phenol oxidase, which is mainly responsible for the degradation of lignin, was significantly higher in the LOW treatment compared to the control and further significantly increased in the HIGH treatment (Table 3; Fig. 3B). However, plant diversity had no significant effects on phenol oxidase activity in any of the exudate treatments (Table 3; Fig. 3B).

## 4 Discussion

The composition and abundances of microorganisms in the rhizosphere are likely to be determined by many different abiotic and biotic factors, such as soil type (Neumann et al., 2014), microclimate (Boone et al., 1998), plant age (Martin, 1971), or plant species identity (Sørensen et al., 2003). Here, we studied the effects of root exudate diversity generated by plant diversity on soil microbial communities. In line with our first hypothesis, soil microbial biomass increased significantly with increasing plant diversity in the control exudate treatment (water only). These findings are consistent with previous studies reporting positive effects of plant diversity on soil microbial biomass (Eisenhauer et al., 2010; Spehn et al., 1994; Steinauer et al., 2009), suggesting that higher availability of plant‐derived resources has a significant and positive effect on soil microbial biomass (Chung, Zak, Reich, & Ellsworth, 2007; Wardle & Nicholson, 2013). Increasing exudate diversity increased total soil microbial biomass mainly in plant monocultures and negated the plant diversity effect. This resulted in similar levels of total microbial biomass across all plant diversity levels in the high‐diversity exudate treatment and confirmed our second hypothesis. Adding easily accessible substrates will subsequently increase soil microbial biomass independently of exudate diversity. Nevertheless, our finding that soil microbial biomass increased successively from the control to the low‐diversity and to the high‐diversity exudate treatments emphasizes the pivotal role of root exudate diversity for total soil microbial biomass. Notably, the low‐ and high‐diversity exudate treatments did only differ in the diversity of compounds but not in their amount; that is, root exudate diversity effects on soil microbial biomass were not due to an increased availability of substrates comparing the low‐ and high‐diversity exudate treatment. As previously assumed (Philippot et al., 2013), we present support for the notion that plant diversity effects on soil microorganisms are related to root exudate diversity of primary metabolites as the exudate diversity treatment negated the plant diversity effect in the present study.

Prior laboratory and field studies in the Jena Experiment (Roscher et al., 2009) assumed that the quality of rhizodeposits rather than plant biomass effects shapes soil microbial community composition (Eisenhauer et al., 2010; Milcu, Partsch, Langel, & Scheu, 2006). In the present study, shoot and root biomass were not affected by plant diversity, supporting the assumption that the quality and diversity of root exudates rather than the sole quantity of substrates affect soil microorganisms.

Analysis of the soil microbial community revealed no significant effects of plant diversity on soil microbial richness. However, the Shannon diversity index increased with higher plant diversity in the control exudate treatment (trend) and at low exudate diversity (significantly) and was similar across all plant diversity levels in the high‐diversity exudate treatment. Further, the soil microbial composition of the control differed significantly to the treatments of diverse exudate cocktails. Moreover, evenness indicated more equally distributed abundances of soil microbial species in the control in comparison with the low‐ and high‐diversity exudate treatments (Fig. 2A,B). Based on the microbial richness and evenness results, we assume that exudate diversity did not increase the number of soil microbial species in the present experiment, but induced alterations in species‐specific abundances and a compositional shift toward fast‐growing copiotrophic soil microorganisms due to a nutrient‐enriched environment (Fierer, Bradford, & Jackson, 2007). Moreover, higher activity of phenol oxidase and cellobiohydrolase at higher exudate diversity indicated increasing dominance of heterotrophic fungi, supporting our assumption of a compositional shift. Former studies found increasing activity of phenol oxidase leading to higher activity of specialized fungi and only a few bacteria like actinomycetes (Keyser et al., 1978; Kirk & Farrell, 1987). Furthermore, basidiomycetes and some ectomycorrhizal fungi are documented to be the most potent producers of cellobiohydrolase (Baldrian & Valásková, 2008; Burke & Cairney, 1998). Nevertheless, to assess compositional shifts of soil microorganisms in more detail, additional analysis and species‐specific identification are needed. As the mycorrhizosphere itself has been shown to impact the associated bacterial community via exudation processes (Mellado‐Vázquez et al., 2016; Scheublin, Sanders, Keel, & van der Meer, 2011), further studies on fungal species might be of great importance.

In line with our third hypothesis, we found higher soil microbial biomass in the carbon‐enriched exudate treatment than in the nitrogen‐enriched exudate treatment. By adding labile C and therefore off‐setting microbial carbon limitation (Kuzyakov & Blagodatskaya, 2015), soil microbial biomass was enhanced. Those findings confirm results of a previous study in the Jena Experiment (Eisenhauer et al., 2010) by showing that soil microbial communities primarily are C‐limited. The root exudate components we used are part of the primary metabolism and their main functions are nutrient sources, chemoattractant signals, and promoters for microbial growth (Haichar, Santaella, Heulin, & Achouak, 2014), which confirms that microorganisms within the rhizosphere used those compounds as an important carbon source (Dennis et al., 2010). Further, when root exudates provide easily decomposable C, enhanced decomposition of soil organic matter may lead to an increase in N availability, reducing N limitation and therefore promoting soil microbial growth (Jones, Hodge, & Kuzyakov, 2004).

Note that we only focus on primary metabolites (no secondary metabolites), and the 18 compounds used in this study do not mimic the root exudate composition of real plants. However, soil microbial biomass within the different diversity levels of exudate cocktails (LOW and HIGH) was significantly different across all plant combinations, indicating that different exudate diversity indeed affects soil microbial biomass and community composition. Nevertheless, more studies on exudate diversity and exudate profile measurements of grassland species are needed to better link plants, root exudates, and soil microbial communities.

In conclusion, we show that significant plant diversity effects on soil microbial biomass and diversity are negated by adding diverse exudate cocktails. Further, root exudate treatments induced variations in abundances of specific microbes and a community shift in microbial community composition. Furthermore, C‐rich exudate cocktails promoted soil microbial biomass more than N‐rich exudate cocktails, suggesting that particularly C‐rich exudates determine the interaction between plant diversity and soil microorganisms in the studied soil. In summary, soil microbial biomass and communities responded to the number of exuded primary metabolites; however, the C/N of root exudates and the resource availability or limitation within the present soil had profound effects on soil microbial biomass. These findings provide the first experimental evidence that root exudate diversity represents a crucial link between plant diversity and soil microorganisms.

## Data accessibility

Data are deposited in the Jena Experiment database.

## Funding Information

Deutsche Forschungsgemeinschaft (‘Ei 862/3‐2’,’FOR 1451’,’FZT 118’).

## Conflict of interest

None declared.
